# Analysis and Functional Prediction of Core Bacteria in the *Arabidopsis* Rhizosphere Microbiome under Drought Stress

**DOI:** 10.3390/microorganisms12040790

**Published:** 2024-04-12

**Authors:** Jianfeng Zhang, Hengfei Zhang, Shouyang Luo, Libo Ye, Changji Wang, Xiaonan Wang, Chunjie Tian, Yu Sun

**Affiliations:** 1 Key Laboratory of Straw Comprehensive Utilization and Black Soil, Conservation College of Life Science, The Ministry of Education, Jilin Agricultural University, Changchun 130118, China; zhangjianfeng06@tsinghua.org.cn (J.Z.); zhanghengfei059868@163.com (H.Z.); yelibo@iga.ac.cn (L.Y.); wangxiaonan1023@163.com (X.W.); 2 Key Laboratory of Mollisols Agroecology, Northeast Institute of Geography and Agroecology, Chinese Academy of Sciences, Changchun 130102, China; luoshouyang@iga.ac.cn (S.L.); wangchangji@iga.ac.cn (C.W.); tiancj@iga.ac.cn (C.T.)

**Keywords:** drought stress, rhizosphere microbiome, core bacteria, *BCP*, *Streptomyces*

## Abstract

The effects of global warming, population growth, and economic development are increasing the frequency of extreme weather events, such as drought. Among abiotic stresses, drought has the greatest impact on soil biological activity and crop yields. The rhizosphere microbiota, which represents a second gene pool for plants, may help alleviate the effects of drought on crops. In order to investigate the structure and diversity of the bacterial communities on drought stress, this study analyzed the differences in the bacterial communities by high-throughput sequencing and bioinformatical analyses in the rhizosphere of *Arabidopsis thaliana* under normal and drought conditions. Based on analysis of α and β diversity, the results showed that drought stress had no significant effect on species diversity between groups, but affected species composition. Difference analysis of the treatments showed that the bacteria with positive responses to drought stress were *Burkholderia-Caballeronia-Paraburkholderia* (*BCP*) and *Streptomyces*. Drought stress reduced the complexity of the rhizosphere bacterial co-occurrence network. *Streptomyces* was at the core of the network in both the control and drought treatments, whereas the enrichment of *BCP* under drought conditions was likely due to a decrease in competitors. Functional prediction showed that the core bacteria metabolized a wide range of carbohydrates, such as pentose, glycans, and aromatic compounds. Our results provide a scientific and theoretical basis for the use of rhizosphere microbial communities to alleviate plant drought stress and the further exploration of rhizosphere microbial interactions under drought stress.

## 1. Introduction

Global warming is accompanied by increases in extreme weather and the frequency of drought and other catastrophic weather events [1]. These sources of water scarcity are further exacerbated by an increasing demand for water due to population growth and economic development [2]. Among abiotic stresses, drought has the greatest impact on soil biological activity and crop yields. Drought increases soil heterogeneity, limits nutrient transfer, increases soil oxygen, reduces microbial biomass, and ultimately reduces crop yields [3,4,5].

The rhizosphere microbiome, which is considered the second plant gene pool [6], has a number of functions that can help alleviate plant drought stress and promote food crop production [7]. Rhizosphere microbes can improve water retention capacity and reduce the water potential of plant roots under drought stress through the formation of extracellular polymeric substances (EPSs) and biofilms [8,9] and the production of proline, which functions as an osmolyte to maintain cell structure and function [10]. Rhizosphere microbes also produce plant hormones that can help plants resist drought [11,12]; indole-3-acetic acid (IAA), cytokinin (CTK), and gibberellin (GA) promote plant growth and stress resistance by increasing biomass [13,14,15], and ethylene (ETH) and abscisic acid (ABA) promote stress resistance by regulating a variety of plant activities [11,12]. Moreover, rhizosphere microbes convert mineral-derived phosphorus and potassium into forms that are easily absorbed by plants [16,17]. Finally, plant-growth-promoting rhizobacteria can improve antioxidant enzyme activity in plants under drought stress [18,19], mitigating the destructive effects of ROS accumulation [20] on structural and functional macromolecules (such as biofilms) and plant homeostasis [21].

The application of rhizosphere microbes to improve plant drought resistance requires an understanding of the effects of drought stress on the structure and diversity of these bacterial communities [22]. A study found that drought induced the enrichment of monoderm bacteria in the rhizosphere microbial community, and it verified the beneficial effect of *Streptomyces* on alleviating plant drought stress through absolute quantification and backcross verification, which may be due to its thicker cell wall peptidoglycan structure [23]. Microbes use their own characteristics to improve plant drought tolerance or accelerate plant recovery, for example by increasing antioxidant enzyme activity [3]. In generations of drought-resistance studies, it has been found that long-term drought accumulation will increase plant adaptation to drought, which may also be the accumulation of drought-resistant bacteria in specific plants [24]. Microbes regulate specific metabolic pathways to assist plants in drought resistance, such as the expression of glycerol-3-phosphate (G3P) uptake genes in the microbiome to assist soybeans in drought stress [25]. This indicates that beneficial bacteria may be enriched to assist plant drought resistance under drought stress. Understanding the effect of drought on the rhizosphere microbial community is of great significance for further study of the mechanism of microbes assisting plant drought resistance.

The recruitment of a high diversity of beneficial microbes is likely key to maintaining the supply of nutrients to plants under drought stress and improving their ecological competitiveness [18,26], but screening microbial determinants of rhizosphere microbial communities requires an understanding of microbe–microbe dynamics under drought stress [27]. Research on beneficial rhizosphere microbes has mostly focused on single strains of rhizosphere growth-promoting bacteria, that is, plant growth promotion [11]. This neglect of microbe–microbe interactions may explain the failure to observe beneficial effects of some rhizosphere growth-promoting bacteria in complex environments, such as field experiments.

In our study, sampling studies were conducted on *Arabidopsis thaliana* rhizosphere soil under drought and normal conditions. The aims of our study were to (i) analyze and compare the composition and structures of the rhizosphere bacterial community of two treatments, (ii) analyze and obtain the core enrichment bacteria under drought stress, and (iii) predict the function of core bacteria, providing a theoretical basis for the study of bacteria for alleviating plant drought. The results may provide a foundation for agricultural utilization and research on microbe–plant interactions.

## 2. Materials and Methods

### 2.1. Plant materials and Drought Stress

**Figure A1:**
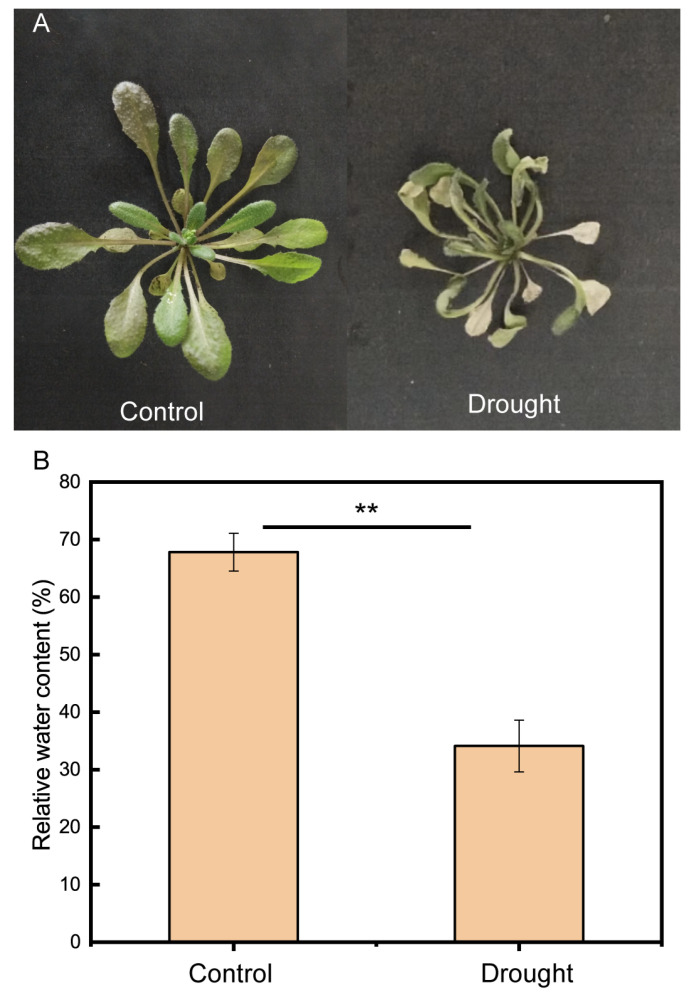
Physiological and biochemical indices of *A. thaliana*. (**A**) Growth status of *A. thaliana* after 14 days of drought and control treatment. (**B**) Relative water content (RWC) of *A. thaliana*. Image shows mean and standard error (*n* = 3 plants). Independent-sample *t* test results showed that the difference was significant, as marked with asterisks (*p* < 0.01). After 14 days of drought treatment and control treatment of *A. thaliana*, the aerial parts were measured.

Columbia wild-type *A. thaliana* was used in this experiment. Twice the number of seeds required for the experiment was soaked in 75% alcohol in a 2 mL sterile centrifuge tube for 10 min, washed three times with sterile water, soaked in 0.1% sodium hypochlorite solution for 10 min, and washed three times with sterile water. Finally, sterile water, which could submerge the seeds, was added to facilitate seeding on plates containing Murashige and Skoog (MS) solid medium. The seeded plates were incubated at 4 °C for two days and then transferred to a climate incubator at 22 °C and 65% relative humidity with a photoperiod of 16 h for 14 days [28,29]. Next, the seedlings were transplanted to planting soil (1:1 (*v*/*v*) moss peat/soil) (pH = 6.59 ± 0.01, EC = 2.58 ± 0.4 mS/cm) in an indoor greenhouse at 25 °C with a photoperiod of 16 h and divided into two treatments: the control treatment and the drought treatment. In the experiment, considering the need for root sampling and the small root system of *A. thaliana*, compound soil was selected. The soil was collected in the experimental field of the Northeast Institute of Geography and Agroecology, Chinese Academy of Sciences, Changchun, Jilin Province (125°406′554″ E, 44°000′361″ N). Moss peat soils (Pindstrup substrate, Ryomgaard, Denmark) were used to enhance soil fertility and water retention and to facilitate soil aeration. Seven days after transplanting, water ceased to be provided to the drought treatment, whereas the control treatment continued to receive water as usual. Fourteen days after watering ended in the drought treatment, differences in plant health between the control and drought treatments were obvious (Appendix A Figure A1A). Finally, the roots of the *A. thaliana* were collected and shaken to remove surface soil and large soil particles. The roots were washed in sterile water and centrifuged at 2000× *g* for 10 min to obtain rhizosphere soil. Aerial parts of the *A. thaliana* were collected to measure relative water content (RWC) by the method of Li et al. [30]. The junction between the aerial parts and roots of *A. thaliana* was carefully cut with scissors, and the whole *A. thaliana* was pulled out after the roots were cut off; the residual roots and soil below the aerial parts of the plant were removed and weighed immediately, and the result was fresh weight (FW). The plants were then soaked in distilled water for 3 h and then taken out and weighed to obtain a saturated weight (SW). The plants were then placed in an oven at 68 °C for 48 h, and their dry weight (DW) was measured when they could be turned into powder by hand. The relative moisture content result was calculated using the following equation:

RWC (%) = (FW − DW)/(SW − DW) ×100%.

The results are shown in an image (Appendix A Figure A1B).

### 2.2. DNA Extraction, PCR, and Sequencing 

DNA was extracted from soil using the FastDNA SPIN Kit For Soil 50 T according to the manufacturer’s instructions, and the DNA concentration was determined by analysis in a NanoDrop spectrophotometer. The V5-V7 hypervariable region of the bacterial 16S rRNA gene was amplified by PCR using the primer pair 799F (5′-AACMGGATTAGATACCCKG-3′) and 1193R (5′-ACGTCATCCCCACCTTCC-3′) in a 10 μL system containing 3 μL of template, 3 μL of 10× buffer, 2.4 μL of dNTPs (2.5 mM each), 0.3 μL of 799F (10 μM), 0.3 μL of 1193R (10 μM), 0.15 μL of HS taq (5 U/μL, from TaKaRa), and 0.85 μL ofddH2O. The temperature program was as follows: initial pre-denaturation at 94 °C for 5 min; 25 cycles of denaturation at 94 °C for 30 s, annealing at 55 °C for 30 s, and extension at 72 °C for 1 min; and a final extension at 72 °C for 5 min [31]. The paired-end sequencing was performed using the Illumina-MiSeq platform (Illumina, San Diego, CA, USA) at the Shanghai Personal Biotechnology corporation. The DADA2 pipeline is used to correct errors in the QIIME2 software (v.2019.4) [32]. First, qiime cutadapt trim-paired was used to remove the primers, and the sequence of the unmatched primers was discarded. Then, the DADA2 pipeline was used to process sequences with primers and barcodes removed. The raw data were filtered by removing the reads with a quality value of less than 20, removing the reads contaminated by the joint, removing the reads containing N, and removing the low-complexity reads (Fast Long Adjustment of Short Reads, v1.2.11) [33]. The DADA algorithm was used to denoise sequences. After denoising, sequences with a single-nucleotide difference were obtained, known as ASVs. To ensure the accuracy of the sequence, the paired-end reads were merged to reconstruct the full amplicon sequence using the overlapping reads to obtain tags in the hypervariable region (the minimum matching length was 15 bp, the overlapping region allowed mismatch rate was 0.1%, and the non-overlapping reads were removed). Finally, singleton ASVs were removed (ASV with a total number of sequences of only 1 in the entire sample was operated by default). After this, the phylogenetic affiliation of each 16S rRNA gene sequence was analyzed by classify-sklearn algorithm [34] using the Silva database (Release132, http://www.arb-silva.de (accessed on 27 November 2012)) [35] of bacteria. For the feature sequences of each ASV, species annotation was performed using a pre-trained naive Bayes classifier in the QIIME2 software. In order to meet the requirement that the samples should be obtained at the same sequencing depth level in subsequent analyses, the ASV abundance tables generated in the previous analyses were flattened. The data were flattened using the rarefaction method. This method predicted the observed ASVs and their relative abundance in each sample by randomly selecting a certain number of sequences from each sample to reach a uniform depth [36,37]. In QIIME2 software, the “qiime feature-table rarefy” function was used to flatten the leveling depth to 95% of the minimum sample sequence size. Data normalization was performed by converting all sample composition data to a sequencing depth of 21,448 per sample. In addition, 0.6 was set as a confidence threshold. 16S rRNA gene amplicon sequencing data are available on NCBI under the Bioproject accession number PRJNA1092351 (https://www.ncbi.nlm.nih.gov (accessed on 28 March 2024)).

### 2.3. Bioinformatics and Statistical Analyses

Taxonomic composition maps at the phylum level were drawn using QIIME2. The Chao1 and Shannon indices for α diversity were calculated by the “phyloseq” package in Microbiome Analyst1. β diversity analysis used principal coordinate analysis (PCoA) to reduce the dimension of microbial data. PCoA was based on the Bray–Curtis distance and used the “ape” package. The Venn diagrams analyzed with the “VennDiagram” package in R (v3.6.1) were used to analyze the species composition of different groups and common unique species. Statistical significance tests of the relative abundance of bacteria between groups were performed using STAMP analysis software (version 2.1.3). LEfSe (LDA effect size), which combined nonparametric Kruskal–Wallis and Wilcoxon rank sum tests with linear discriminant analysis (LDA) effect size, analyzed bacteria with significant differences between groups through the “Python LEfSe” package and R’s “ggtree” package. In order to further confirm the species that have a positive response to drought stress, a Circos diagram was used to further characterize the dominant strains under drought treatment. The Circos diagram of the relationship between samples and species at the genus level was constructed using the genescloud platform (https://www.genescloud.cn (accessed on 20 March 2024). To further analyze the interactions between microbes and the locations of different microbes in the network diagram, a co-occurrence network diagram was constructed. Pairwise Spearman correlation coefficients (Spearman’s |R| ≥ 0.6, *p* < 0.05) were used to analyze the co-occurrence network. Network topology features were visualized and computed with Gephi (v0.10.1). The R scripts used for producing co-occurrence networks shown in this manuscript are provided as Supplementary Materials. To analyze the differences in bacterial functional groups between treatments, PICRUSt2 was used to predict the functions of the rhizosphere microbial community. Tests of differences between groups were performed by one-way analysis of variance (Tukey’s multiple comparison test) (SPSS statistical software v20.0). Differences were considered statistically significant at *p* < 0.05.

## 3. Results

### 3.1. Bacterial Community Diversification and Overall Species Composition

**Figure 1:**
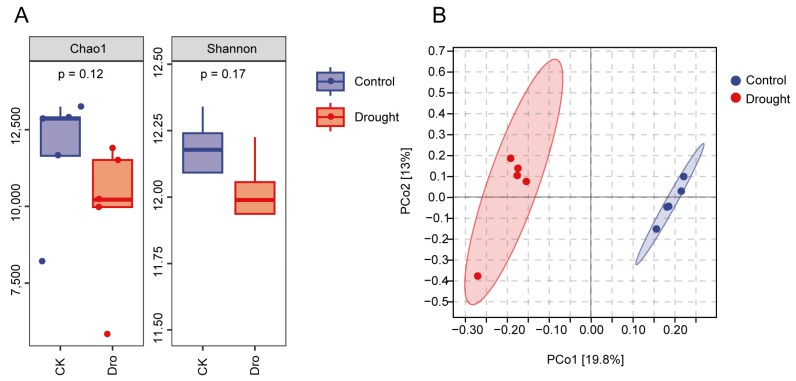
Analysis of bacterial diversity in the *Arabidopsis* rhizosphere. (**A**) The Chao1 index and Shannon index show α diversity and richness at the ASV level. (**B**) Principal coordinate analysis (PCoA) of bacteria in the *Arabidopsis* rhizosphere based on the Bray–Curtis distance and unweighted UniFrac matrix at the ASV level. Each dot represents a sample. Blue dots correspond to the normal watering treatment (control), and the red dots correspond to the drought treatment.

To determine whether drought stress affects *Arabidopsis* rhizosphere bacteria, comparative analyses were performed at the ASV level. The diversity and richness of the *A. thaliana* rhizosphere bacterial communities did not differ significantly between the control and drought treatments. However, the Chao1 and Shannon indices (Figure 1A) showed that rhizosphere diversity and richness tended to decrease in the drought treatment compared with the control. Principal coordinate analysis (PCoA) based on the Bray–Curtis distance at the ASV level (Figure 1B) showed complete separation of the bacterial communities in the control and drought treatments along the first principal axis, indicating that rhizosphere bacterial composition differed significantly between the two treatments (the confidence ellipse includes 95% of the data). These results suggest that drought stress mainly affected the composition of the bacterial community in the rhizosphere of *A. thaliana*.

**Figure 2:**
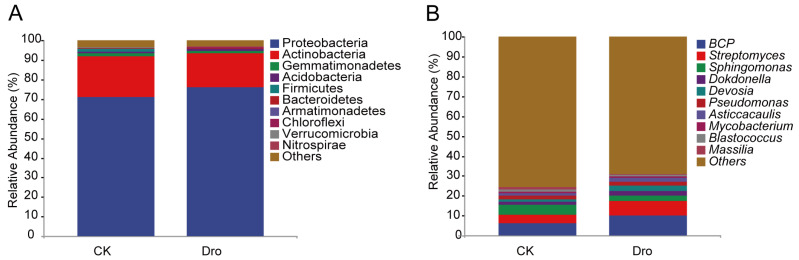
The average relative abundances of the top 10 bacterial phyla (**A**) and genera (**B**) in the rhizosphere of *A. thaliana*. CK, normal watering (control); Dro, drought treatment.

To further explore the differences in *A. thaliana* rhizosphere bacterial community composition, the top ten bacterial phyla were compared (Figure 2A). In both treatments, Proteobacteria (71.04–76.18%) and Actinobacteria (17.27–20.86%) were the predominant phyla and together represented 91.90–93.44% of all sequences, indicating that the distribution of bacteria in the *A. thaliana* rhizosphere was relatively concentrated at the phylum level. At the genus level (Figure 2B), *Burkholderia-Caballeronia-Paraburkholderia* (*BCP*) (6.29–10.09%), *Streptomyces* (4.08–7.18%), and *Sphingomonas* (4.96–2.69%) were the predominant genera. The top ten bacterial genera represented a smaller proportion of sequences in the control treatment (31.75%) than in the drought treatment (36.48%), indicating greater dispersion of rhizosphere bacterial community composition at the genus level under normal growth of *A. thaliana*, which may be more conducive to community stability.

### 3.2. Analysis of Differential Abundance between Treatments

**Figure 3:**
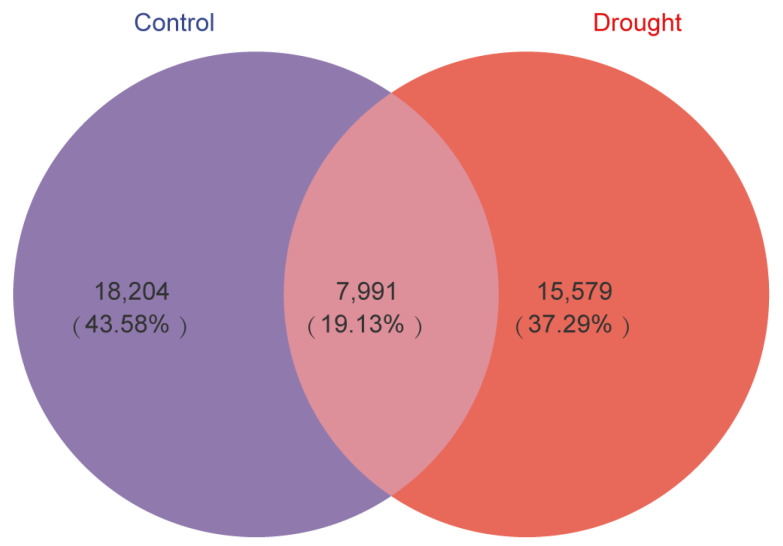
Venn diagrams of bacterial ASVs in the *Arabidopsis* rhizosphere in the control (purple) and drought (red) treatments. The area of overlap indicates ASVs shared by the treatments. The number in each fraction indicates the number of ASVs.

The Venn diagram in Figure 3 compares the bacterial ASVs detected in the rhizosphere of *A. thaliana* in the control and drought treatments. Only 19.3% of the ASVs were shared between the treatments, indicating that rhizosphere bacterial community composition differed greatly between the treatments, even though the difference in species diversity between the treatments was not significant. In addition, the total number of ASVs decreased by 2625 (10.02%) in the drought treatment compared with the control.

**Figure 4:**
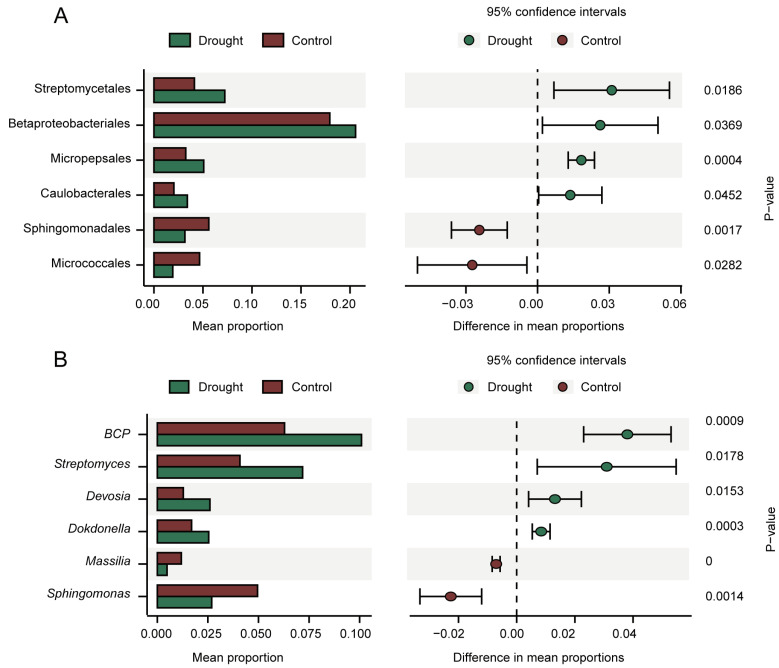
STAMP analysis of differences in relative abundance at the order (**A**) and genus (**B**) levels (95% confidence intervals, *p* < 0.05). The bar charts on the left show the difference in order or genus abundance between the treatments. The dot bar graphs on the right show the differences in order or species abundance between the treatments.

STAMP analysis was performed to analyze the differences in rhizosphere bacterial community composition at the order (Figure 4A) and genus (Figure 4B) levels. The orders Streptomycetales, Betaproteobacteriales, Micropepsales, and Caulobacterales were enriched under drought stress, whereas Sphingomonadales and Micrococcales were enriched in the control. Six of the top ten genera differed significantly in relative abundance between the treatments, of which four were enriched under drought stress: *BCP*, *Streptomyces*, *Devosia*, and *Dokdonella*. Although these differences were observed at the relative abundance level and are not absolutely quantitative, enrichment suggests a positive response to drought stress.

**Figure 5:**
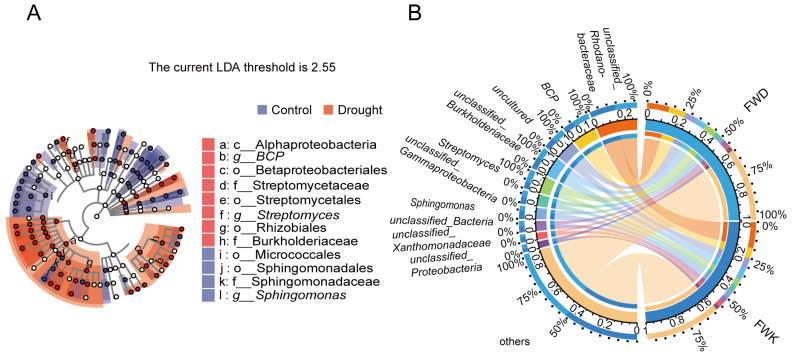
Analysis of marker species. LEfSe analysis of marker species (**A**). Taxonomic branch diagram of the taxonomic hierarchy of the main taxa from phylum to genus (inner to outer) in the community. The node size corresponds to the average relative abundance of the classification unit; hollow nodes represent taxa with insignificant differences between treatments, whereas the colored nodes indicate taxa that are significantly more abundant in the treatment with the corresponding color. The Circos diagram of the relationship between samples and species on the genus level (**B**). The right semicircle represents the species composition in the sample, the color of the outer band represents the group from which it comes, the color of the inner band represents the species, and the length represents the relative abundance of the species in the corresponding sample. The circle on the left represents the distribution ratio of species in different samples at the genus level, the outer ribbon represents species, the inner ribbon represents different groups, and the length represents the distribution ratio of the sample in a certain species.

LEfSe analysis was performed to evaluate differences in all taxonomic levels simultaneously. The taxonomic branch diagram (Figure 5A) shows the taxonomic levels of the main groups in the sample community from phylum to genus (inside to outside). Bacterial groups with LDA scores of 4 were identified as significantly enriched and were labeled in the taxonomic branch diagram (Figure 5A). Compared with the drought treatment, the control was significantly enriched in Micrococcales and *Sphingomonas* (from order to genus). By contrast, drought stress significantly enriched Rhizobiales (from class to order), *BCP* (from order to genus), and *Streptomyces* (from order to genus). When combined with the results of STAMP difference analysis, these results indicate that the core microbial groups enriched by drought stress were *BCP* and *Streptomyces*.

To determine whether *BCP* and *Streptomyces* groups respond positively to drought, a Circos diagram (Figure 5B) of species relationships was used for analysis. Based on the top 10 species with relative abundance at the genus level, the dominant bacterial groups enriched under drought stress were *BCP* and *Streptomyces*. The dominant group of the control was *Sphingomonas*. *BCP* and *Streptomyces* were proven to have a positive response to drought stress.

### 3.3. Co-Occurrence Network Analysis

**Figure 6:**
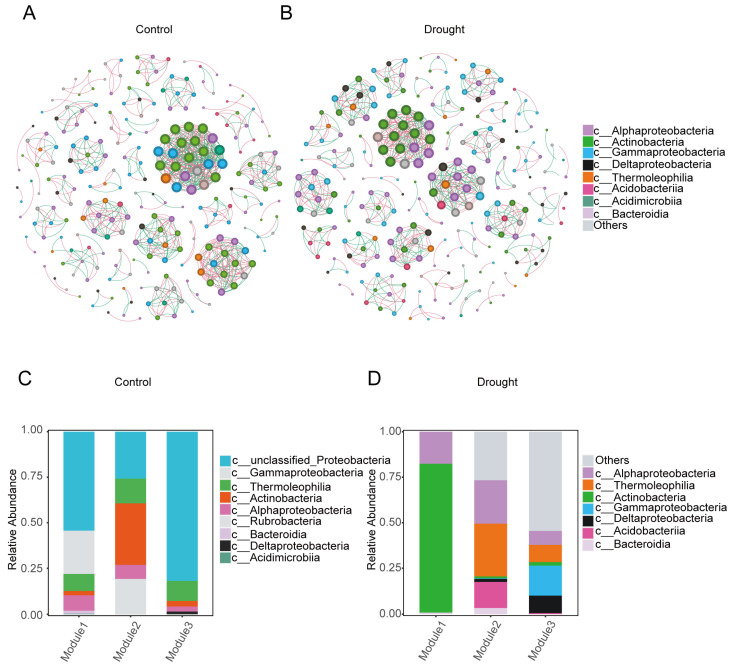
Co-occurrence network analysis of the bacterial community in the rhizosphere of *A. thaliana.* (**A**) Normal watering (control) treatment. (**B**) Drought treatment. Each node in the network represents a bacterial population. Different node colors at the class level represent different main classes, and the size of the node represents the degree of connection of each ASV. Pink connections between nodes indicate positive correlations, whereas green connections indicate negative correlations. (**C**,**D**) The species composition of the three largest modules in the normal watering (control) treatment (**C**) and drought treatment (**D**) networks.

To further explore the positions of the core differential bacterial groups in the whole bacterial co-occurrence network, a bacterial co-occurrence network diagram (Spearman’s |R| ≥ 0.6, *p* < 0.05) (Figure 6A,B) and table (Table 1) were established at the class level. In the control, the rhizosphere bacterial co-occurrence network contained 242 nodes and 782 connections, of which 54.99% were positive connections and 45.01% were negative connections. In the drought treatment, the number of connections decreased to 682, of which 55.28% were positive and 44.72% were negative. The increased isolation of bacteria due to the physical conditions imposed by drought may have reduced bacterial interconnections and the possibility of competition. In summary, drought stress reduced the complexity of the bacterial co-occurrence network (average degree: control, 3.231; drought, 2.795).

The degree of modularization in the network diagram was higher in the drought treatment than in the control. Actinobacteria, Alphaproteobacteria, Gammaproteobacteria, and Thermoleophilia were predominant in the three modules of the control (Figure 6C), whereas Actinobacteria and Alphaproteobacteria were predominant in the bacterial network under drought stress (Figure 6D). These results indicate that drought stress reduced the stability of the bacterial community in the rhizosphere of *A. thaliana*.

**Table 1:** Analysis of bacterial community co-occurrence networks.

Sample	Nodes	Total Edges	Positive Edges	Negative Edges	Average Degree
Drought	244	682	377 (55.28%)	305 (44.72%)	2.795
Control	242	782	430 (54.99%)	352 (45.01%)	3.231

**Figure 7:**
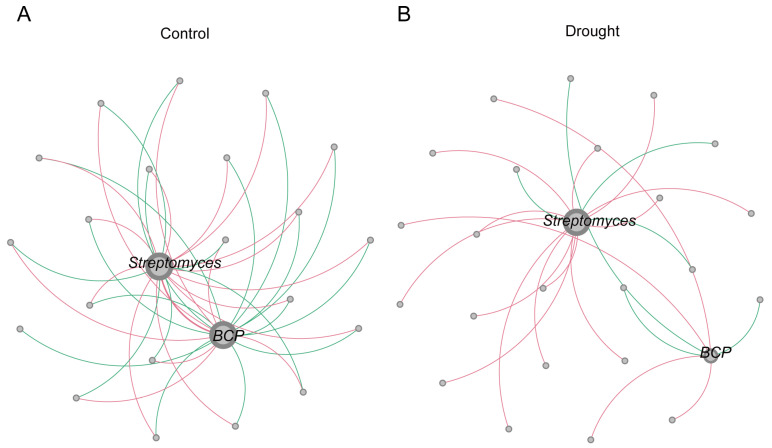
Co-occurrence network analysis of the *Streptomyces* and *BCP* in the (**A**) normal watering (control) treatment and (**B**) drought treatment. Each node in the network represents a bacterial population. The size of the node represents the degree of connection of each ASV. Pink connections between nodes indicate positive correlations, whereas green connections indicate negative correlations.

The changes in the core rhizosphere bacteria under drought stress were analyzed by comparing the network diagrams of the treatments. For both treatments, *Streptomyces* was in the largest module in the network diagram (Figure 7). *BCP* was in the largest module in the control (Figure 7A), and its network connectivity decreased significantly under drought stress (Figure 7B). The number of negative links in the network diagram of *BCP* decreased from 15 in the control to 3 under drought stress, whereas the number of positive links decreased from 7 to 4. These results suggest that the enrichment of *BCP* under drought stress reflected a decrease in competitors. The increase in *Streptomyces* from the control to the drought treatment indicates a positive synergetic link in the dominant position. Therefore, compared with *BCP*, *Streptomyces* was more active in the adaptation of the rhizosphere bacterial community to drought stress.

### 3.4. Functional Prediction of the Bacterial Communities

**Figure 8:**
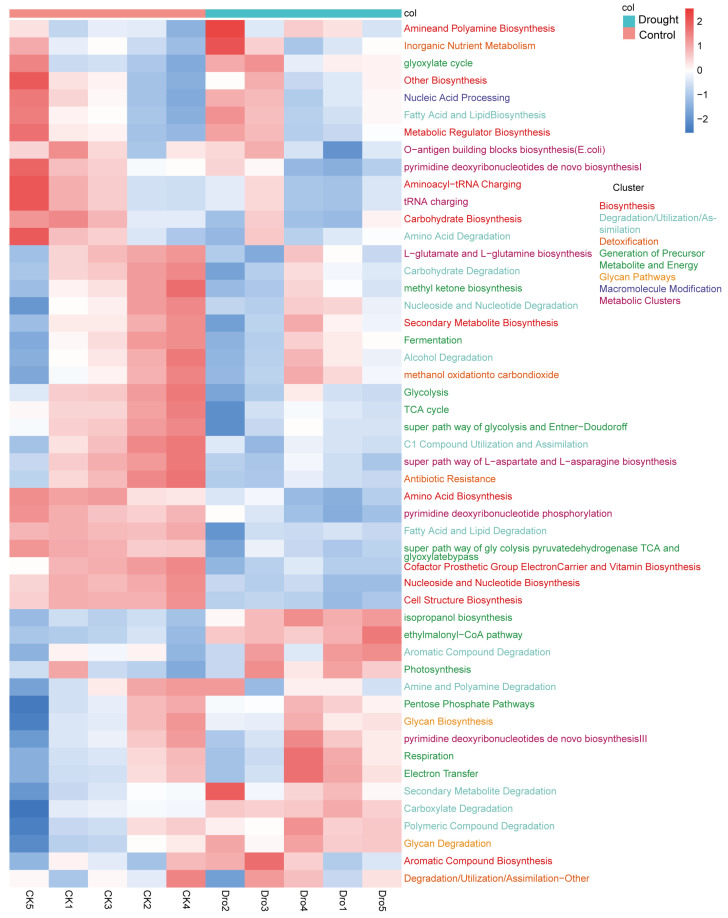
Heatmap of the relative abundance of metabolic pathways in the rhizosphere bacterial community of *A. thaliana*. Each small grid represents a metabolic pathway, and its color represents the treatment in which relative abundance is higher. The higher the relative abundance, the deeper the color. Each column presents the expression of the metabolic pathway in a specific sample.

**Figure 9:**
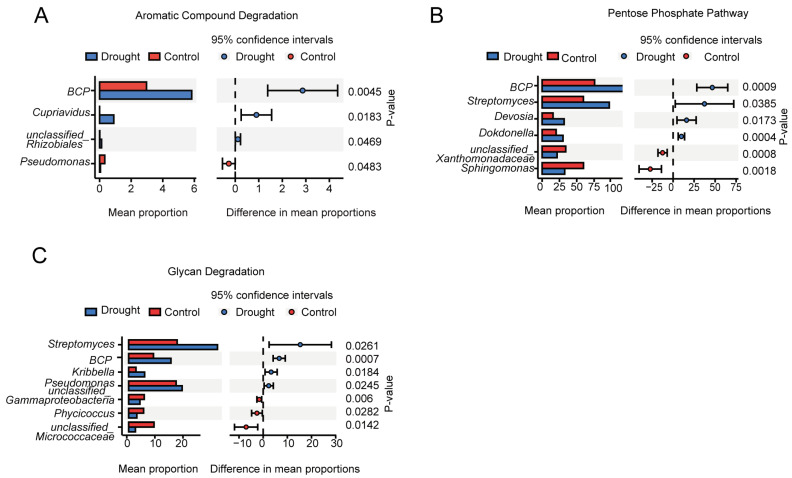
Functional prediction of the bacterial community in the rhizosphere of *A. thaliana*. Pathways with significant differences in relative abundance between the control and drought treatments included (**A**) aromatic compound degradation, (**B**) the pentose phosphate pathway, and (**C**) glycan degradation.

**Figure A2:**
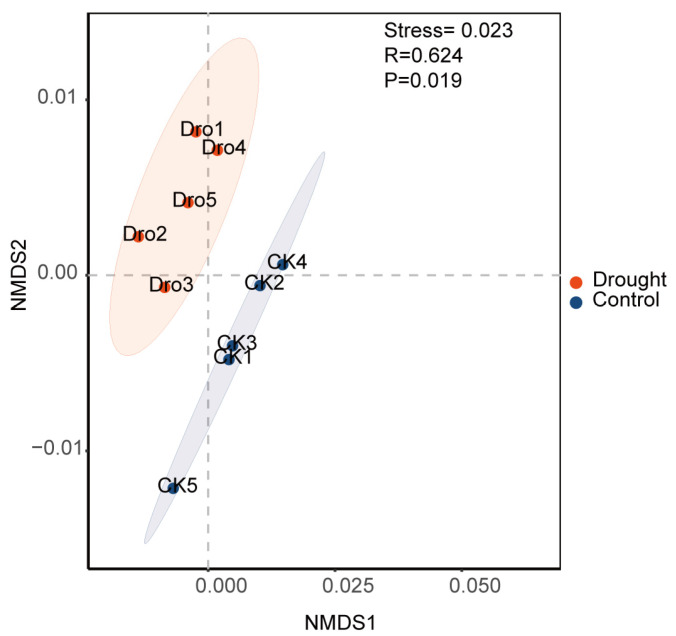
Functional prediction for the bacterial community in the rhizosphere of *Arabidopsis thaliana*.

PICRUSt2 was used to predict the functions of the rhizosphere bacterial community. PICRUSt2 is based on non-metric multidimensional scaling (NMDS), which, similar to PCoA, simplifies the data structure by reducing the dimension of the sample distance matrix. However, unlike PCoA, NMDS is not affected by the sample distance and considers only size relationships. Thus, for data with complex structures, the ranking results of NMDS may be more stable than those of PCoA. The NMDS ordination showed a significant separation of the functional composition of the bacterial community between the drought and control treatments (stress = 0.023; r = 0.624; *p* = 0.019) (Appendix A Figure A2). An analysis of the abundances of genes encoding enzymes showed that nitrogenase, catalase, and 1-aminocyclopropane-1-carboxylate (ACC) deaminase were significantly increased under drought stress, further supporting the positive role of rhizosphere bacteria in plant drought resistance. Heatmap cluster analysis of the drought response (Figure 8) revealed increases in rhizosphere bacterial functions related to the metabolism of a wide range of carbohydrates, including pentose, glycans, and aromatic compounds, suggesting an increase in the range of utilizable carbon substrates. Compared with the drought treatment, the control treatment had more functions related to bacterial survival, such as biosynthesis, generation of precursor metabolites and energy, detoxification, cofactors, prosthetic groups, electron carriers, vitamin biosynthesis, nucleoside and nucleotide biosynthesis, cell structure biosynthesis, TCA cycle, fermentation, and antibiotic resistance. To further explore the core bacteria in the functional modules of drought enrichment, three metabolic pathways of polysaccharide degradation were selected for STAMP species composition difference analysis: aromatic compound degradation (Figure 9A), the pentose phosphate pathway (Figure 9B), and glycan degradation (Figure 9C). This analysis indicated that the enrichment of these metabolic pathways in the drought treatment compared to the control was associated with significant increases in the relative abundances of *BCP* and *Streptomyces*.

## 4. Discussion

Drought stress can negatively affect plant health and yield [24,38,39]. Harnessing the positive effects of the rhizosphere microbial community on plant drought resistance requires an understanding of the effects of drought stress on the structure and diversity of the bacterial communities [22]. Drought stress has been shown to reduce root microbial biomass [3,4,40] and root microbial diversity and richness [23,41]. However, we did not observe a significant difference in the α diversity of rhizosphere bacteria between the drought and control treatments; only β diversity differed significantly. This discrepancy may reflect the fact that few bacterial groups underwent large changes; most bacterial groups had only small responses [42]. As a result, there were significant differences in the relative abundances of specific groups between the treatments, even though the difference in diversity was not significant [4]. In general, Proteobacteria are the predominant rhizosphere bacteria [23], consistent with the predominance of the phyla Proteobacteria and Actinobacteria observed here. Venn diagram analysis of the changes in *A. thaliana* rhizosphere bacteria under drought stress showed that only 19.13% of species were shared between the two treatments, which may reflect the combined response of the plant, soil, and microorganisms to drought [23,41]. In line with the α-diversity and β-diversity results, drought altered the composition of the bacterial community in the rhizosphere of *A. thaliana*.

A study on prolonged drought in the same plot found that drought increased the accumulation of specialized bacteria that were used to alleviate drought stress in plants [24]. Therefore, we studied the enrichment of rhizosphere bacteria in *A. thaliana* under drought stress and analyzed its position in the rhizosphere bacterial community structure. STAMP analysis of group differences showed that *BCP*, *Streptomyces*, *Devosia*, and *Dokdonella* were significantly enriched in response to drought. Wang et al. [43] studied the ability of *BCP* to alleviate drought stress in *Atractylodes lancea*. Xu et al. [23] found that *Streptomyces* was enriched in the rhizosphere of sorghum under drought conditions and indicated that this enrichment may be beneficial to plants. The specific roles of *Devosia* and *Dokdonella* under drought stress have not been detailed. To further explore the core bacteria in the rhizosphere of *A. thaliana* under drought stress, all classification levels of rhizosphere bacteria were analyzed simultaneously by LEfSe, which identified *BCP* and *Streptomyces* as marker bacteria. These results were verified by co-occurrence network analysis.

Bano et al. [44] previously identified core rhizosphere microbes by fitting a network model. We found that the core bacteria were generally associated with key functions. PICRUSt2 prediction of the functions of the rhizosphere bacterial community showed that the core bacteria were significantly related to polysaccharide degradation and utilization [45,46].

Studies of drought stress have focused extensively on *Streptomyces*, which is significantly enriched in the rhizosphere of plants under drought stress [23,47]. *Streptomyces* produces IAA and siderophores to help plants alleviate drought stress [48,49,50]. For example, Chukwunemeet al. [49] studied the ability of *Streptomyces* to promote plant growth and found that it produces IAA; Xu et al. [50] found that applying exogenous iron to plants under drought stress eliminated drought-induced actinobacterial enrichment. *Streptomyces* also contributes to plant drought resistance by affecting the expression of plant genes. Li et al. [51] found that inoculation of wheat with *Streptomyces* increased the expression levels of several drought-resistance genes, such as EXPA6, EXPA5, P2CS, and SnRK12, and Abbasi et al. [48] determined that *Streptomyces* enhanced the drought resistance of tomato by regulating the expression of the transcription factors ERF1 and WRKY70, which inhibit tomato growth under drought stress.

We found that *Streptomyces* plays an important role not only in plant–microbe interactions but also in the stability of rhizosphere bacterial community structure under drought stress. The rhizosphere bacterial co-occurrence network diagram showed that *Streptomyces* was in the core module in the control and drought treatments. This prominence may be because *Streptomyces* facilitates signal exchange with the microbial community. Krespach et al. [52] found that an arginine ketone (arginoketide) polyketide produced by *Streptomyces* mediates its cross-border interactions with fungi. As a chemical signal, arginine is a general component of the microbial communication network that shapes microbial communities. Wang et al. [53] found that antibiotics can be used as signals between different *Streptomyces* to regulate microbial population interactions. Communication between microorganisms is undoubtedly an important element of microbiome dynamics [27].

*BCP* are mostly plant endophytes [54] and can alleviate nutritional and abiotic stress in plants. For example, Wang et al. [43] found that *BCP* and associated rhizosphere microorganisms alleviate the drought stress of *A. lancea* and promote the accumulation of its medicinal components. In *Rhododendron* and *Atractylodes*, *BCP* can reduce damage caused by high-temperature stress [55,56]. Kim et al. [57] found that plant roots exude lactone, a rhizosphere signal molecule for plant–microbe interactions, into the rhizosphere when plant nutrition is deficient. Lactone promotes the recruitment of rhizosphere microorganisms to help plants absorb nutrients from the soil. A microbial analysis of 16 rice varieties showed that *BCP* was significantly correlated with lactone content in root exudates, providing new insights into the interactions between plants and rhizosphere microbes [57]. Plant-growth-promoting effects of *BCP* have also been observed. *BCP* alleviates high-temperature stress and promotes root growth in *A. lancea* and soybean [55,58]. In the present study, *Streptomyces* and *BCP* were the predominant genera, which was reflected in the predominant phyla. In the co-occurrence network diagram of *Arabidopsis* rhizosphere microbes in the control, both *Streptomyces* and *BCP* were in the largest module. In the drought treatment, *Streptomyces* remained in the core module, but the connectivity of *BCP* decreased significantly. In the drought treatment, the performance of *BCP* was similar to that of *Sphingomonas*, except that *BCP* maintained high relative abundance under drought stress. Thus, *BCP* may be a drought-tolerant microorganism. At the same time, due to the limitation of the relative abundance of microbiome data, the correlation in the co-occurrence network (using a metric Spearman’s correlation) can be affected by this and show spurious correlations [59]. In order to solve this problem, further attention should be paid to the verification of the drought-resistance effect in the future when core bacteria are returned to *A. thaliana*.

Drought can directly or indirectly affect the soil carbon cycle [58,60]. Drought not only reduces the stability of soil organic carbon (SOC) by disrupting macroaggregates and altering soil chemical properties but also enhances the conversion of refractory C by changing the composition and activity of the microbial community [58]. We also found that drought stress increased the utilization of complex organic matter by rhizosphere bacteria. *Streptomyces* has been reported to utilize complex carbon sources, including pesticides and insecticides. *Streptomyces* has high polysaccharide-degradation potential [61] and can degrade cellulose and chitin [62,63]. The carbon cycle in the soil microbial community is controlled by several groups of common bacteria [64]. Stone et al. [45] found that *Bradyrhizobium*, *Acidobacteria RB41*, and *Streptomyces* together account for 45–57% of carbon flow through bacteria. *BCP* may act as a biological probiotic by degrading autotoxins produced by continuous cropping [46,65]. *BCP* also plays an important role in the degradation of the pesticide acephate and can use pesticides and their degradation products as sole sources of C, N, and P for growth [66]. This may explain the potential complex carbon source utilization ability of *BCP* found in this study.

## 5. Conclusions

In this study, we found that (1) drought stress affected the composition and structure of plant rhizosphere bacterial communities; (2) drought stress reduced the complexity, connectivity, and stability of the rhizosphere bacterial network of *A. thaliana*; and (3) *Streptomyces* and *BCP* were identified as active markers of drought stress. In the co-occurrence network analysis, it was found that *Streptomyces* and *BCP* were at the core of the rhizosphere bacterial community. In the PICRUSt2 analysis, they were found to be enriched functions associated with the utilization of complex organic matter. These results provide a foundation for exploring rhizosphere microbe–microbe interactions under drought stress and a basis for developing rhizosphere microbiomes to alleviate the effects of drought on crops.

## Data Availability

Data are contained within the article.

## Supplementary Materials

The following supporting information can be downloaded at: https://www.mdpi.com/article/10.3390/microorganisms12040790/s1, R’s co-occurrence network.
